# Whole-genome analysis and mutation pattern of SARS-CoV-2 during first and second wave outbreak in Gwangju, Republic of Korea

**DOI:** 10.1038/s41598-022-14989-y

**Published:** 2022-07-05

**Authors:** Shilpa Chatterjee, Choon-Mee Kim, You Mi Lee, Jun-Won Seo, Da Young Kim, Na Ra Yun, Dong-Min Kim

**Affiliations:** 1 Department of Biomedical Science, College of Medicine, Chosun University, Gwangju, Republic of Korea; 2 Premedical Science, College of Medicine, Chosun University, Gwangju, Republic of Korea; 3 Department of Internal Medicine, College of Medicine, Chosun University, 588 Seosuk-dong, Dong-gu, Gwangju, 501-717 Republic of Korea

**Keywords:** Infectious diseases, Viral infection

## Abstract

To investigate the specific genomic features and mutation pattern, whole and near-complete SARS-CoV-2 genome sequences were analyzed. Clinical samples were collected from 18 COVID-19–positive patients and subjected to nucleic acid purification. Cell culture was performed to extract various SARS-CoV-2 isolates. Whole-genome analysis was performed using next-generation sequencing, and phylogenetic analyses were conducted to determine genetic diversity of the various SARS-CoV-2 isolates. The next-generation sequencing data identified 8 protein-coding regions with 17 mutated proteins. We identified 51 missense point mutations and deletions in 5′ and 3′ untranslated regions. The phylogenetic analysis revealed that V and GH are the dominant clades of SARS-CoV-2 circulating in the Gwangju region of South Korea. Moreover, statistical analysis confirmed a significant difference between viral load (P < 0.001) and number of mutations (P < 0.0001) in 2 mutually exclusive SARS-CoV-2 clades which indicates frequent genomic alterations in SARS-CoV-2 in patients with high viral load. Our results provide an in-depth analysis of SARS-COV-2 whole genome which we believe, can shed light in the understanding of SARS-COV-2 pathogenesis and mutation pattern which can aid in the development of prevention methods as well as future research into the pathogenesis of SARS-CoV-2 and therapeutic development.

## Introduction

Coronavirus disease 2019 (COVID-19) is caused by a single-stranded, nonsegmented, positive-sense, enveloped RNA virus named severe acute respiratory syndrome coronavirus 2 (SARS-CoV-2), which belongs to the family Coronaviridae, genus Betacoronavirus^1^. SARS-CoV-2 was first isolated in China at the Wuhan fish market in December 2019. As a global pandemic, COVID-19 has spread rapidly to 215 countries and infected almost 269 million people, with 5.36 million reported deaths (as of 18th December, 2021). In South Korea, approximately 560,000 cases and 4644 deaths have been reported (as of 18th December, 2021) (www.worldometers.info/coronavirus).

SARS-CoV-2 is the single-stranded RNA virus with a genome size of ~ 29.9 kB in length^2^. The full-length SARS-CoV-2 RNA genome contains 5′ untranslated region (*UTR*), various open-reading frames (*ORFs*), spike (*S*) gene, envelope (*E*) gene, membrane (*M*) gene, nucleocapsid (*N*) gene, 3′UTR, several unidentified non-structural ORFs and a poly (A) tail^3^. The ORF1ab is the largest SARS-COV-2 genes among which ORF1a gene encodes for polyprotein pp1a, contains 10 nonstructural proteins (NSPs) and ORF1b gene encodes for polyprotein pp1ab (contains 16 NSPs)^3^. The 3′UTR contains 4 structural proteins: *S*, *E*, *M*, and *N* proteins and 8 accessory genes^3^. The structural *S* protein contains a variable receptor-binding domain (RBD), which has provided insights into frequent variation in the genomic sequences of SARS-CoV-2^4,5^.

Evaluating the number and rate of mutation of RNA viruses, although challenging, is necessary to trace the evolutionary relationship. Generally, RNA viruses have up to a million times higher mutation rates, than do DNA viruses^6^. According to GISAID data, approximately 23.6 mutations per year are identified in the SARS-CoV-2 sequences, but there is no evidence regarding the functional effects of these mutations on viral replication^7^. So far number of variants (variants of concern) have been identified around the world such as B.1.1.7^8^, B.1.351^9^, P.1^10^, and B.1.617^11^ where major mutations were observed in the receptor binding domain of the spike protein. This is a well-established fact that over time viruses undergo genetic drift due to selection, resulting in a number of predominant variants that provide a challenge to any pandemic response. Although SARS-CoV-2 spreads rapidly, very little is known about the transmissibility and pathogenicity of the virus^12^. Therefore, thorough investigation and monitoring of the genetic sequences of SARS-CoV-2 are necessary to track its evolution; these data can reveal distinct epidemiological characteristics of the pathogen and should yield sufficient information for the design of therapeutics and vaccines^13,14^.

Several studies have already shown the mutation and viral-RNA replication pattern of SARS-CoV-1, but the presence of notable differences between SARS-CoV-1 and SARS-CoV-2 makes it impractical to predict a correlation between SARS-CoV-2 viral load and the number of mutations in the whole-genome sequence^15^. Hence, further in-depth studies of SARS-CoV-2 are required to understand the epidemiology, transmissibility, virus shedding, evolution, and disease outcomes.

To address this issue, we performed a whole-genome analysis of 32 clinical samples isolated from COVID-19-positive patients with diverse severity of the disease. The objective of this study was to understand molecular genetic evolutionary features, evolution patterns and differences between the first and second wave isolates in Gwangju area of ROK. Though we performed this study on small dataset collected from single geographical location of ROK, we believe that our findings can shed light in the understanding of SARS-COV-2 etiology and mutation pattern which can aid in the development of prevention methods as well as future research into the pathogenesis of SARS-CoV-2 and therapeutic development.

## Methods

### Ethics statement

The study protocol was approved by the Institutional Review Board (IRB) of Chosun University (IRB approval number: CHOSUN 2020-04-003-002. 2020-02-120) and all methods were performed in accordance with the relevant guidelines and regulations. Written informed consent was obtained from the patients. A copy of the written consent is available for review by the Editor of this journal up on request.

### Collection of patient samples and viral RNA extraction

Upper and lower respiratory tract clinical samples (nasopharyngeal and oropharyngeal swabs were obtained using the UTM™ Kit containing 1 mL of a viral transport medium as well as sputum and plasma samples of COVID-19 patients were collected. All patients were admitted to the Chosun University Hospital at the time of sample collection and all the multiple isolates were collected across different time points. The clinical samples were collected at two time-points: the first wave of the pandemic from February to May 2020 (11 isolates) and the second wave from June 2020 to April 2021 (21 isolates). After collection, all samples were subjected to viral nucleic acid extraction using the Real-prep Viral DNA/RNA Kit (Biosewoom, South Korea). The RNA was quantified by the Korea Centers for Disease Control and Prevention (KCDC) method and using a PowerChek 2019-nCoV Real-time Kit (Kogene Biotech, Seoul, South Korea).

### Real-time reverse-transcription polymerase chain reaction (RT-qPCR)

RT-qPCR was performed by targeting the RNA-dependent RNA polymerase (RdRp) gene and the E gene. A total of 20 µL of the reaction mixture (master mix) was prepared by adding 5 µL of the template RNA, 11 µL of the One-Step RT-PCR Premix included in the PowerChek 2019-nCoV Real-time Kit (Kogene Biotech, Seoul, South Korea), and 4 µL of each primer and probe mixture. An appropriate positive control (internal control) was used for each PCR run. A primer/probe mixture (for RdRp and E), and a positive control template (for RdRp and E) are included in the PowerChek 2019-nCoV Real-time PCR Kit. The primer and probe sequences are kept confidential. The thermal cycling protocol comprised reverse transcription at 50 °C for 30 min, followed by reverse transcription inactivation at 95 °C for 10 min, and then 40 cycles of 95 °C for 15 s and 60 °C for 1 min (7500 instrument; Applied Biosystems, Foster City, CA, USA). All of the samples were tested twice.

### The cell culture study for isolating SARS-CoV-2 variants from COVID-19 patients

This procedure was performed to isolate SARS-CoV-2 from infected Vero E6 cells (African green monkey kidney cells, Korean Cell Line Bank, KCLB No. 21587). Monolayers of Vero E6 cells were inoculated with each PCR-positive respiratory sample. Dulbecco’s modified Eagle’s medium (DMEM; Gibco, Thermo Fisher Scientific, USA) was used to maintain the infected cells. The medium was supplemented with 2% fetal bovine serum and penicillin–streptomycin solution (Gibco, Thermo Fisher Scientific Inc., USA). Briefly, 200 µL of the 100 penicillin-streptomycin solution was added to 500 µL of the respiratory samples (nasopharynx or oropharynx swab, sputum, or saliva) from COVID-19 patients and incubated at 4 °C for 1 h. The mixtures were vortexed every 15 min, centrifuged at 3000 rpm for 20 min, and 200 µL of the supernatants were applied to Vero E6 cells, to infect them. After 5 days of incubation at 37 °C in a 5% CO_2_ incubator, cytopathic effects (CPEs) were observed under a microscope at 24-h intervals for up to 7 days and a cell suspension was prepared by scraping the cells. After two passages, RT-qPCR was performed to determine the C_t_ and CPEs to confirm virus replication.

### Next-generation sequencing (NGS) and phylogenetic analysis

To confirm each SARS-CoV-2 mutation, NGS was performed for whole-genome analysis. NGS was performed by the Illumina NovaSeq 6000 or Illumina MiSeq method (Illumina, San Diego, CA), which produced 150-base pair-end reads per sample. The isolated viral RNA was subjected to whole-genome sequencing. Nine clinical samples and 23 viral-particle–containing cell culture supernatants were used for the whole-genome analysis. NGS libraries were prepared by means of the data obtained from the sequences, and the analysis was performed via barcode-tagged sequencing technology (Celemics Inc., Seoul, Korea). Multiple-sequence comparison by log-expectation was conducted to perform the multiple-sequence alignment of SARS-CoV-2 genomes. Phylogenetic tree was constructed by the maximum likelihood (M-L) method^16^ using Molecular Evolutionary Genetics Analysis across Computing Platforms (MEGA X) software^17^. One thousand bootstrap replicates were utilized to evaluate replicated-tree confidence.

### Mutation analysis

The complete or near-complete SARS-CoV-2 genome in the isolates from each of the 18 enrolled patients was analyzed to identify mutations in the protein-coding sequences and to compare the sequences with the reference SARS-CoV-2 genome (hCoV-19/Wuhan-Hu-1/2019) retrieved from the Global Initiative for Sharing All Influenza Data (GISAID, https://www.gisaid.org/) database. All amino acid sequences were aligned with the reference sequence (NCBI ID: NC_045512) using the NCBI BLAST protein alignment tool. Further, the wave-wise number and frequency of mutation per sample were calculated.

### Statistical analysis

The number of mutation and viral loads between the two COVID-19 waves were compared for each clinical sample, and a two-tailed student t-test was performed to evaluate the differences. The null hypothesis was that there were significant differences in the number of mutation and viral load between the two groups. The median, interquartile range and probability (P) values were calculated for both, the nucleotide and amino acid mutations and the viral load for each sample in both periods (V clade in the first wave and GH clade in the second wave). Statistical significance was defined as a P value of less than 0.05, for all statistical analyses. GraphPad Prism version 7.0 was used for this analysis.

## Results

### Demographic distribution

In this study, we analyzed the whole genomes of SARS-CoV-2 in 32 clinical isolates collected from 18 patients who were admitted to Chosun University Hospital, Gwangju, South Korea. SARS-CoV-2 infection was detected in patients aged 29–93 years, and the prevalence was 33.3% among patients aged 21–50 years and 66.6% among those aged > 50 years. The data on sample collection and details of patients’ symptoms are presented in Table 1.

**Table 1:** Data on patient characteristics, sample types, symptoms at collection, clades, and number of mutations found in the SARS-CoV-2 genomes isolated from COVID-19 patients.

Patient (age/sex)	Sample type	Symptoms at sample collection	Symptom severity	Clade/lineage		Mutations	
				GISAID	Nextstrain	Nucleotide	Amino acid
M/46	Nasopharynx	Coughing, chills	Mild to moderate	V	19A	7	5
M/30	Nasopharynx	Coughing, sore throat, chills	Mild to moderate			5	4
	Sputum					4	3
M/30	Sputum	Febrile sensation	Mild to moderate			5	3
	Nasopharynx					5	3
F/29	Nasopharynx	Sore throat, myalgia, chills	Mild to moderate			6	3
	Nasopharynx					5	3
	Sputum					6	3
	Nasopharynx					5	3
	Nasopharynx					6	3
	Nasopharynx					6	4
M/68	Nasopharynx	Febrile sensation, chills, fever	Mild to moderate	GH	20C	12	6
F/53	Nasopharynx	Myalgia, fever	Mild to moderate			12	7
F/81	Nasopharynx	Coughing, rhinorrhea	Mild to moderate			16	11
M/68	Sputum	Chills, fever	Severe			20	13
M/76	Sputum	Fever, cough, dizziness, chills	Mild to moderate			14	8
M/74	Sputum	Cough, phlegm	Mild to moderate			21	9
F/51	Sputum	Sore throat	Mild to moderate			20	8
F/37	Nasopharynx	Asymptomatic	Asymptomatic			24	15
M/83	Nasopharynx	Hypotension, low oxygen saturation	Critical/fatal			19	7
	Nasopharynx					19	7
	Cell supernatant					20	9
	Cell supernatant					20	8
	Plasma				20A	17	9
	Nasopharynx				20C	17	7
	Sputum					17	9
M/82	Nasopharynx	Fever	Mild to moderate			22	12
	Nasopharynx					22	12
F/93	Cell supernatant, nasopharynx	Myalgia	Mild to moderate			15	10
F/66	Cell supernatant, sputum	Dyspnea	Mild to moderate			22	10
F/78	Cell supernatant, sputum	Sore throat, myalgia, chills	Mild to moderate	GH	20C	22	9
F/87	Cell supernatant, nasopharynx	Hypotension, low oxygen saturation	Critical/fatal			20	7

### Analysis of the various SARS-CoV-2 isolates

Clinical samples were inoculated into the Vero E6 cells for virus isolation. CPEs were examined under a microscope at 24-h intervals for up to 1 week. Results were designated as negative if no CPE was observed within seven days. Viral RNA from the culture supernatant was subjected to RT-qPCR analysis, targeting the RdRp and E genes. Samples with a C_t_ < 35 for both target genes were designated as positive for SARS-CoV-2 and subjected to whole-genome analysis (Supplementary Table 1). Relative viral copy number for RdRp and E genes were calculated from plasmid DNA containing complete gene using ten-fold serial dilutions (1 × 10^9^ to 1 × 10^1^ copies/µL).

### Phylogenetic analysis

**Figure 1:**
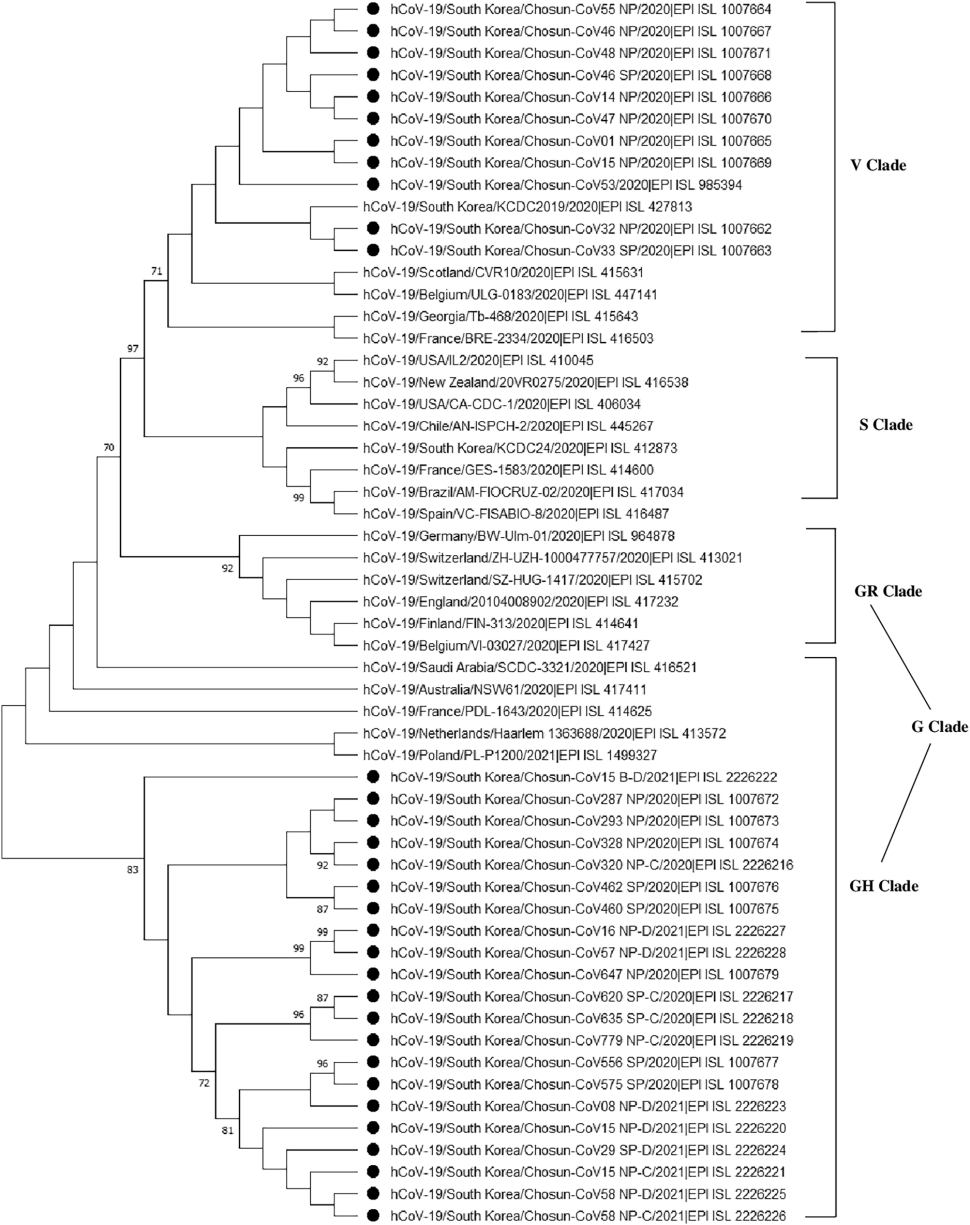
Phylogenetic analysis of complete/near complete genomes of the severe acute respiratory syndrome coronavirus 2 (SARS-COV-2) isolates. The evolutionary tree was constructed by the Maximum Likelihood (M-L) method using MEGA X software. The tree is drawn to scale, with branch lengths reflecting the number of substitutions per site (indicated below the branches). Only bootstraps greater than 70 are shown. To evaluate replicated tree confidence, 1000 bootstrap replicates were performed. The evolutionary distances were computed using the Tamura-Nei method and have been presented in the units of number of base substitutions per site. This analysis involved 56 nucleotide sequences. Clades are indicated on the right.

The phylogenetic analysis was performed for 32 different SARS-CoV-2 sequences under study and several reference sequences obtained from the GISAID database (Fig. 1). Two major clades, V (*ORF3a*-G251V) and GH (*S*-D614G and *ORF3*-Q57H) were identified by the phylogenetic analysis based on point mutations in the clinical samples.

### Mutation analysis of SARS-CoV-2 whole genomes

**Figure 2:**
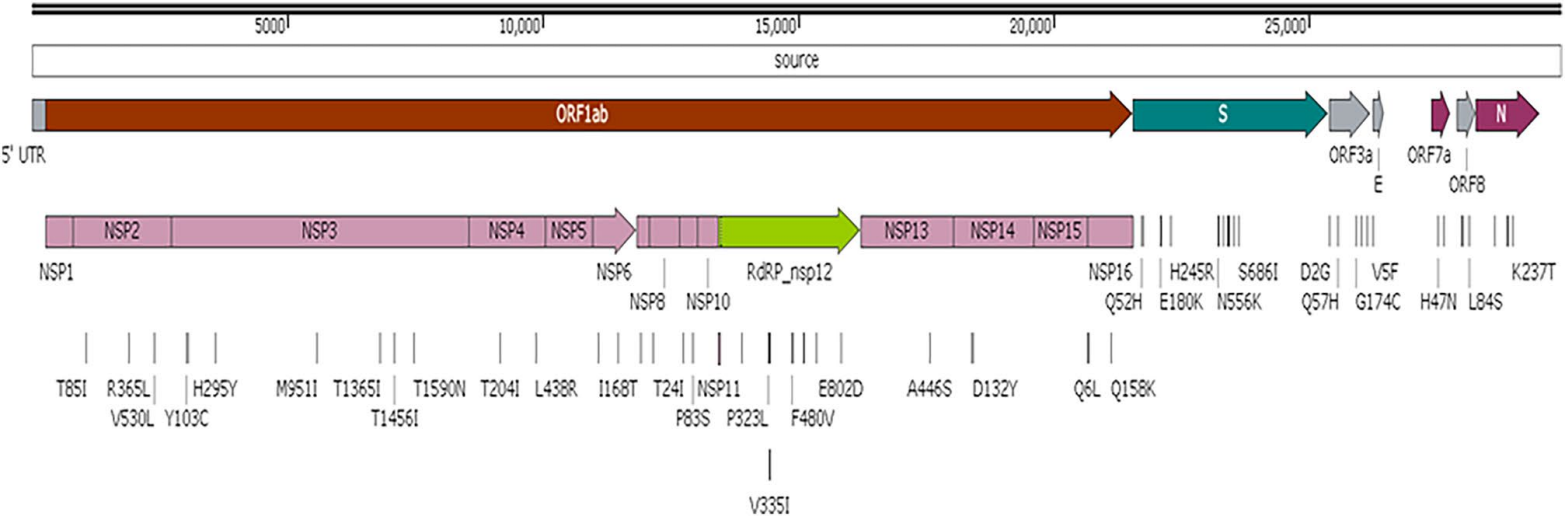
Schematic mapping of the mutations in the SARS-CoV-2 whole genome. The full-length (29,903 bp) SARS-CoV-2 *Betacoronavirus* RNA genome consists of an ORF1a encoding 10 nonstructural proteins (nsp1–10) and an ORF1b encoding 16 nonstructural proteins (nsp1–16) in the 5´ untranslated region (UTR). The structural proteins correspond to 4 genes in the 3′ UTR: spike (*S*), envelope (*E*), membrane protein (*M*), and nucleocapsid (*N*) genes. The mutations and changes in amino acid residues (with position numbers) have been individually presented in the diagram.

Whole genomes of the 32 isolates were analyzed next. Eight protein-coding regions (*ORF1a, ORF1b, S, ORF3, E, ORF7a, ORF8,* and *N*) with 17 mutated proteins were identified and analyzed to detect new/rare mutations and distinct clades of SARS-CoV-2. Compared to the reference sequence (NC_045512), the results of NGS revealed 51 nonsynonymous substitutions (i.e., point mutations), including 27 amino acid mutations in the nsp-coding region (*ORF1a* and *ORF1b*) and 24 amino acid mutations in the structural-protein-coding region (*S, ORF3, E, ORF7a, ORF8*, and *N*) (Fig. 2). Clinical samples were collected from patients during the 2 waves of COVID-19 and the significance of the variation in the mutations was analyzed. The NGS data revealed that point mutations (substitutions) were most common among the Gwangju isolates of SARS-CoV-2. Additionally, deletions were identified in both the 5′ and 3′UTR. We identified clade V (*ORF3a*-G251V) and clade GH (*S*-D614G, *ORF3*-Q57H) from 11 and 21 samples, respectively, of the 32 sequences tested in this study (Table 1).

Substitutions were most frequently observed in the *ORF1ab* region (nsp1–16; nucleotide positions 266–21,555). The common amino acid mutations were M951I (in *NSP3* gene) and L37F in *NSP6* gene (during the first wave: 11 samples) as well as T85I (in *NSP2* gene), S25L (in *NSP7* gene), P323L (in *NSP12* gene), and Q6L in *NSP16* gene region (during the second wave: 21 samples). In addition to these major substitutions, several other mutations were found in the *ORF1ab* region. Notably, mutations M951I and L37F occurred with the G251V (*ORF3*) mutation among the samples collected during the first wave, which mostly corresponds to clade V. For clade GH, during the second wave, mutations in the *ORF1ab* gene (T85I, S25L, P323L, and Q6L) occurred with those in the (D614G) *spike* and (Q57H) *ORF3* genes. The D614G *Spike* mutation and P323L *NSP12* mutation are defining characteristics of the G clade (Pangolin clade B.1, the parent of all four of the major circulating Variants of Concern). In our study, NGS data revealed that all of these second wave samples bear the expected signature of the dominant clade. Thirty-eight additional point mutations were identified in different protein-coding regions. In the *NSP2* region, 3 distinct point mutations were detected: R365L (EPI_ISL_2226217 and EPI_ISL_2226218), V530L, and T531P (EPI_ISL_2226224). During the second wave, 4 novel mutations were detected in the *NSP12* region that codes for SARS-CoV-2 *RdRp*: D153Y (EPI_ISL_1007679), P323L (21 samples), V335I (EPI_ISL_2226222), and L638F (EPI_ISL_1007675 and EPI_ISL_1007676). In *NSP16*, along with the common mutation Q6L, one distinct mutation (Q158K; EPI_ISL_2226222) was identified.

Only single point mutation was found in the envelope (*E*) gene (V5F; EPI_ISL_1007674) and in the spike (*S*) region (A623S; EPI_ISL_2226222) in the second-wave samples. Two mutations were identified in the *ORF7a* (H47N and P84S) and *ORF8* regions (P30S and P36S). Three new mutations—G120E, T205I, and K237T—were identified in the nucleocapsid (*N*) protein-coding region. In addition to the substitutions, a number of deletions were identified in few genomic regions, mainly in the 5′ UTR (*NSP14* in *ORF1ab*). Deletions were found mainly in 3 whole genomes: EPI_ISL_2226225 (between positions 19,298 and 19,474: *NSP14*), EPI_ISL_2226222 (between positions 7006 and 7035: *NSP3*; 27,533–27,554: ORF7a), and EPI_ISL_2226224 (between positions 6887 and 7036: *NSP3*; 8422–8598: NSP3–4; 19,298–19,549: *NSP14*; and 20,125–20,173: *NSP15*). Supplementary Tables 2 and 3 summarizes all the mutations, together with the positions, amino acid residues, number of isolates/patients in which those mutations were observed at different time point.

### Wave-wise identification of frequency of SARS-COV-2 mutations

**Figure 3:**
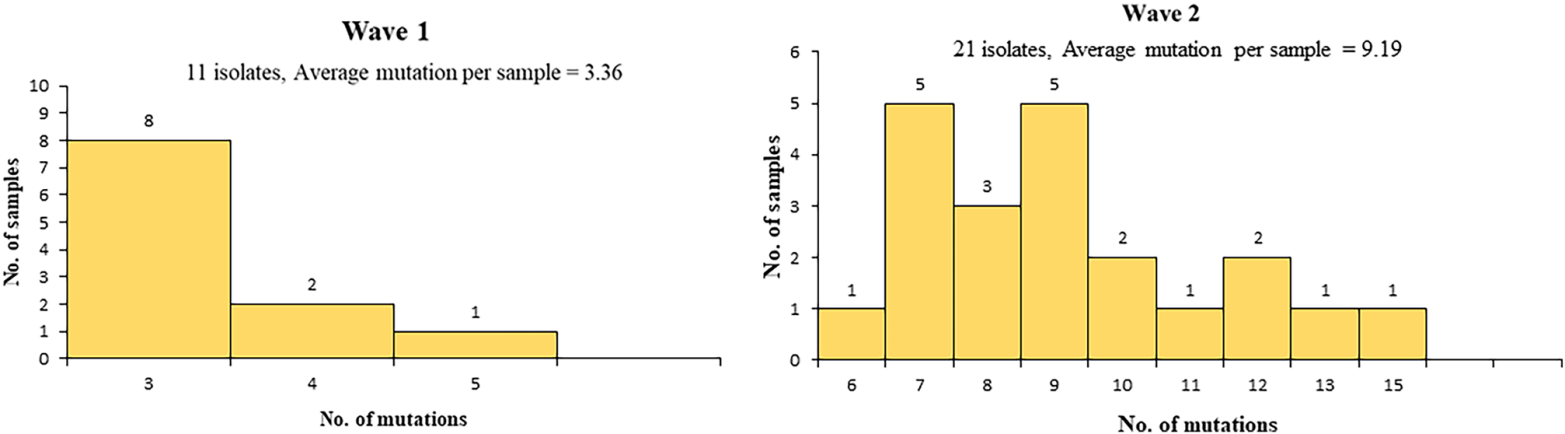
Graph representing the number of mutations per sample for wave 1 and wave 2. The average mutations per sample observed in wave 1 is 3.36 for 11 isolates and in wave 2 is 9.19 for 21 isolates.

**Figure 4:**
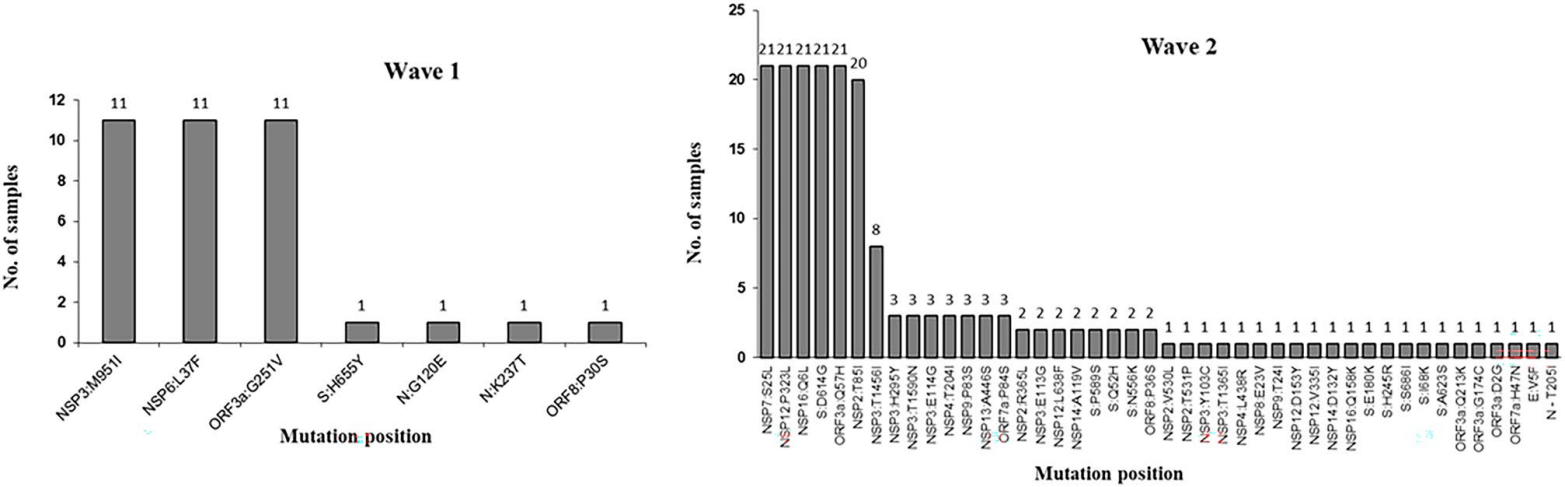
Graph representing the number of protein level alteration observed in first and second wave samples.

In comparison to the reference sequence, all the study sequences from both waves showed multiple mutations. The average number of mutations observed in each sample in wave 2 was 9.19 which is significantly higher than wave 1(average 3.36 mutation per sample) (Fig. 3). Further, we analyzed the number of protein level alteration in wave 1 and wave 2 isolates that also revealed the non-identical mutation pattern in both waves. In wave 1, the most frequently observed mutations were NSP3:M951I, NSP6:L37F, and ORF3a:G251V and in wave 2 NSP7:S25L, NSP1:P323L, NSP16:Q6L, S:D614G, ORF3a:Q57H, NSP2:T85I, and NSP3:T1456I were the most dominant mutations which was not observed in wave 1 (Fig. 4). The statistical analysis using t-test confirmed that in patients with a higher viral copy number and low C_t_, the number of mutations increased over time (from the first to second wave). In a comparative analysis, statistically significant differences (P < 0.0001 and P < 0.001) were observed between the number of mutations and viral load for the two clades (Table 2) which revealed the significant genomic and proteomic differences in the SAR-COV-2 viral isolates between both waves.

**Table 2:** Statistical analysis showing significant difference between viral load and number of mutation in the two clades from the clinical samples of the patients with COVID-19.

	Mutation						Viral load					
	Nucleotide			Amino acid			E gene			RdRp gene		
	Median	IQR*	p-value**	Median	IQR	*p-value*	Median	IQR	*p-value*	Median	IQR	*p-value*
V clade (n = 11)	5	1	< 0.0001	3	1	< 0.0001	1.59E + 07	6.25E + 07	< 0.001	3.42E + 07	8.72E + 07	< 0.001
GH clade (n = 21)	20	5		9	3.5		6.13E + 08	7.50E + 09		8.98E + 08	7.60E + 09	

*Interquartile range.

**Probability value.

## Discussion

The ongoing pandemic, caused by the rapid spread of the novel coronavirus to 215 countries, has already affected approximately 171 million people worldwide^18^. The effective strategies, such as thorough testing, quarantine, and awareness programs that were adopted by the South Korean government have helped contain the spread of infection, leading to a markedly low death rate (approximately 1.4%)^19^. The present situation serves as an example of how infectious diseases can be dangerous, in terms of deaths and damage to health. Compared to past pandemics, the present situation is advantaged by the unparalleled abundance of advanced scientific and technical means to combat COVID-19.

To understand the dynamic nature of SARS-CoV-2 infection, analysis of whole-genome sequences is crucial. The rapidly evolving characteristics of the virus also make it necessary to analyze the whole genome; this research will improve our understanding of how fatality, infectivity, and pathogenicity are associated with each mutation^20^. Here, we analyzed 32 complete or near-complete genome sequences of SARS-CoV-2, extracted from the clinical samples of 18 COVID-19 patients; the samples were collected at 2 time-points (2 waves of COVID-19). The SARS-CoV-2 could replicate in other cells (Caco-II cells), in addition to Vero E6 cell. The first SARS-COV-2 was successfully isolated from human airway epithelial cells^21^. Since human airway epithelial cells require 4–6 weeks to differentiate in vivo, despite being the non-human primate cell line, for routine SARS-COV-2 virus culture, this cell line is being used which provides a good basis for research studies showing faster viral replication in Vero E6 cells^22,23^.

Phylogenetic analysis of the substitutions in the 32 isolates, revealed 2 major clades: V (*ORF3a*-G251V) and GH (*S*-D614G and *ORF3*-Q57H). The 11 isolates of clade V (most prevalent from February to May 2020 in Gwangju) were found to constitute sister lineages with one another and showed phylogenetic similarity with the GISAID reference strains. Two isolates (EPI_ISL_1007662 and EPI_ISL_1007663) were found to form sister lineage with the Korean strain EPI_ISL_427813 and formed a cluster with the strains reported in Scotland (EPI_ISL_415631) and Belgium (EPI_ISL_447141). The remaining 21 isolates, (GH clade, mostly corresponding to the period from June 2020 to April 2021) form sister lineages with one another and the GH clade, based on the GISAID reference sequences. According to the GISAID global clade classification of SARS-CoV-2, 8 major clades have been identified: S, O, L, V, G, GH, GR, and GV^24^. Based on this classification, we found the V clade (11 isolates) clusters during early 2020, at the beginning of the pandemic, and the GH clade (21 isolates) clusters during the second wave, after June 2020. According to Nextstrain, there are 11 major clades or lineages (19A, 19B, and 20A–20I), which have been used to track SARS-CoV-2^25^. Here, we identified 2 major lineages, 19C and 20C (including 11 first-wave isolates and 20 s-wave isolates, respectively), and 1 minor lineage (20A; EPI_ISL_2226222).

Analyzing the whole genome of SARS-CoV-2 is of utmost importance for tracking its increased transmissibility and its potential to alter virulence^26^. In our study, we analyzed the whole genome of SARS-CoV-2 to detect novel or rare substitutions (i.e., point mutations). In the 5′UTR, major amino acid substitutions were observed in *ORF1ab* (*NSP1*–16). The most common new substitutions identified in nsp include T85I in *NSP2*; E114G, H295Y, M951I, and T1456I in papain-like protease (*NSP3*); L37F in replicase (*NSP6*); S25L in primase (*NSP7*); and Q6L in methyltransferase (*NSP16*) (Supplementary Table 2). In addition, a common substitution (P323L) was found in the *RdRp* region (*NSP12*) of 21 isolates^25^. Furthermore, 14 substitutions were identified in the *ORF1ab* region. Worldwide, several distinct nonsynonymous point mutations have been detected in *ORF1ab*^27^.

Moreover, the novel mutations in *NSP2* and *NSP3* that were identified might alter the infectivity of SARS-CoV-2, thereby leading to changes in its proofreading function and pathogenesis^28^. Additionally, the nsp6 protein of SARS-CoV-2 favor viral replication; therefore, mutations in nsp6 can alter viral autophagy^29^. The different substitutions in the amino acid sequences of *NSP7* and *NSP16* can affect genome replication and evasion from host cell immunity^30–32^.

Several other point mutations in the structural protein region at the 3′UTR were detected in our study. Among the 21 second wave samples, the most common mutations identified were D614G in the *S* protein and Q57H in *ORF3*. The D614G mutation in the *S* protein region increases the infectivity of the virus and diminishes the neutralizing activity of the serum samples from convalescents^33^. The G251V mutation in *ORF3* was detected in the 11 first-wave isolates. According to Alexander et al., such a substitution in the amino acid residue directly correlates with the increased fatality rate of SARS-CoV-2^34^.

In the 5′UTR, deletions were detected in *NSP3, NSP4, NSP14*, and *NSP15* in the *ORF1ab* region. SARS-CoV-2 *ORF1ab* plays an important role in the proteolytic processing of NSP1–16 that functions in the viral infection cycle, and its alteration may affect this cycle^35^. Yang et al. have described the importance of the 5′UTR for the whole-genome regulatory function of the coronaviruses^36^. Ma et al. have reported that *NSP14* serves as a proofreading exoribonuclease and is required for viral replication^37^.

In the 3′UTR, deletions were detected in *ORF7a*, which may change the viral immunomodulatory activity and alter immune cell binding^38^. Additionally, several other point mutations were identified in the structural protein regions that may modify the virus ability to bind host cell receptors and neutralizing antibodies as well as host cell apoptosis, protein synthesis in the cell, and the transmissibility of the virus particles. Furthermore, a few rare mutations, such as G174C, Q213K, and D2G, were identified in *ORF3* (*NS3*). These mutations are more prevalent in Europe, Asia, and North America^39^. Several new substitutions in the *E*, *ORF7a, ORF8*, and *N* regions were also identified (Supplementary Table 2).

It is a well-established fact that the transmission capacity of SARS-CoV-2 is much higher than that of other viruses of the *Coronaviridae* family^40^. The increase in mutation frequency of G614 from March (26% of GenBank sequences) to May (75% of GenBank sequences) further proves the altered transmissibility of SARS-CoV-2^41^. Therefore, it is necessary to monitor the changes in viral load and alterations in genome sequences associated with it. Accordingly, we statistically analyzed the relationship between the number of mutations and viral copy number, and observed a wave-wise statistically significant difference between the two. These data are suggestive of frequent genomic alterations in patients with a high viral load and lower RT-qPCR Ct. This result was supported by our finding that the D614G variant has high transmissibility due to enhanced replication fitness in primary epithelial cells (upper respiratory tract cells), which is directly linked to a high viral titer and disease severity^42,43^.

Overall, the whole-genome analysis helped us identify specific substitutions in different regions of the SARS-CoV-2 genome. A total of 51 nonsynonymous point mutations was identified in various protein-coding regions, including 27 in the 5′UTR and 24 in the 3′UTR. Several rare mutations were detected in 8 protein-coding regions, particularly, in *ORF1ab*, *RdRp, ORF3a, S*, and *ORF7a*. Alteration of the amino acid sequences encoded by these regions might directly affects the viral proofreading, pathogenicity, replication, and transmissibility. The prevalence of these mutations varies greatly based on geographical location and changes over time. In this study, we observed a significant rise in the number of mutations in each isolate with low Ct value in wave 2 than wave 1 which is quite similar to the pattern observed in SARS-CoV-2 pandemic waves in Hiroshima, Japan^44^. Although both waves observed in Gwangju area had multiple number of mutational changes over time but wave 2 tended to have a higher number of genomic alterations.

This study has few limitations that includes small clinical sample subset (only 18 enrolled patients) used within a single geographical location of ROK. Therefore, further research using big population pool is necessary to validate the findings and understand the broader scenario.

## Conclusions

This study identified a number of previously unreported nonsynonymous mutations and revealed significant genomic and protein level differences in the SARS-COV-2 isolates between the first and second wave. The observed statistically significant differences between viral load and the number of mutations indicates frequent genomic alterations in SARS-CoV-2 in patients with a high viral load. Thus, in future studies, an in-depth analysis with big dataset along with the sequencing of the SARS-CoV-2 whole genome should be performed to elucidate the transmission dynamics and to design effective treatment strategies to counter the further spread of this virus.

## Supplementary Information

Supplementary Tables.

## Data Availability

We shared our data to figshare with 10.6084/m9.figshare.14936013. Furthermore, all the mentioned isolates in Supplementary Table 1 are newly generated sequences from this study and the sequence information can be referred to as GISAID accession number.
