# Supplementary material for: Whole-genome analysis and mutation pattern of SARS-CoV-2 during first and second wave outbreak in Gwangju, Republic of Korea
Source: Sci Rep. 2022 Jul 5;12:11354. doi: 10.1038/s41598-022-14989-y (PMC9255444; doi:10.1038/s41598-022-14989-y)
Supplement: Supplementary file 1 — Supplementary Tables. [file 41598_2022_14989_MOESM1_ESM.docx]

**Supplementary Table 1: Viral copy numbers and C_t_ in 32 clinical samples from COVID-19 patients as determined via the analysis of the *E* and *RdRp* genes**

|  |  | **E Gene** | | **RdRp** | |
| --- | --- | --- | --- | --- | --- |
| **Isolates** | **Sample**  **type** | **C_t_ value** | **Viral Copy** | **C_t_ value** | **Viral Copy** |
| EPI_ISL_985394 | Sputum | 13.76 | 1.46E+09 | 14.17 | 9.79E+08 |
| EPI_ISL_1007662 | Nasopharynx | 14.09 | 1.17E+09 | 15.47 | 4.86E+08 |
| EPI_ISL_1007663 | Sputum | 18.53 | 5.41E+07 | 18.7 | 8.55E+07 |
| EPI_ISL_1007664 | Nasopharynx | 32.31 | 3.90E+03 | 31.8 | 7.43E+04 |
| EPI_ISL_1007665 | Nasopharynx | 33.45 | 1.77E+03 | 35.15 | 1.23E+04 |
| EPI_ISL_1007666 | Nasopharynx | 18.35 | 6.11E+07 | 18.7 | 8.56E+07 |
| EPI_ISL_1007667 | Nasopharynx | 18.25 | 6.55E+07 | 18.51 | 9.47E+07 |
| EPI_ISL_1007668 | Sputum | 27.31 | 1.24E+05 | 26.98 | 9.95E+05 |
| EPI_ISL_1007669 | Nasopharynx | 20.3 | 1.59E+07 | 20.4 | 3.42E+07 |
| EPI_ISL_1007670 | Nasopharynx | 22.21 | 4.23E+06 | 21.22 | 2.20E+07 |
| EPI_ISL_1007671 | Nasopharynx | 23.78 | 1.43E+06 | 24.02 | 4.88E+06 |
| EPI_ISL_1007672 | Nasopharynx | 10.99 | 9.96E+09 | 12.4 | 2.53E+09 |
| EPI_ISL_1007673 | Nasopharynx | 14.26 | 1.04E+09 | 15.5 | 4.78E+08 |
| EPI_ISL_1007674 | Nasopharynx | 17.41 | 1.17E+08 | 18.98 | 7.35E+07 |
| EPI_ISL_1007675 | Sputum | 17.44 | 2.84E+07 | 17.06 | 3.95E+07 |
| EPI_ISL_1007676 | Sputum | 14.04 | 2.50E+08 | 13.74 | 3.25E+08 |
| EPI_ISL_1007677 | Sputum | 13.68 | 1.55E+09 | 11.99 | 3.15E+09 |
| EPI_ISL_1007678 | Sputum | 12.64 | 6.13E+08 | 12.14 | 8.98E+08 |
| EPI_ISL_1007679 | Nasopharynx | 14.63 | 1.72E+08 | 14.16 | 2.49E+08 |
| EPI_ISL_2226216 | Nasopharynx | 11.23 | 8.42E+09 | 10.3 | 7.85E+09 |
| EPI_ISL_2226217 | Sputum | 11.48 | 7.09E+09 | 10.26 | 8.01E+09 |
| EPI_ISL_2226218 | Sputum | 31.69 | 5.98E+03 | 30.63 | 1.39E+05 |
| EPI_ISL_2226219 | Nasopharynx | 35.26 | 5.07E+02 | 33.51 | 2.96E+04 |
| EPI_ISL_2226220 | Nasopharynx | 8.49 | 5.62E+10 | 8.65 | 5.03E+10 |
| EPI_ISL_2226221 | Nasopharynx | 11.37 | 7.67E+09 | 10.15 | 8.52E+09 |
| EPI_ISL_2226222 | Plasma | 9.94 | 8.73E+09 | 9.92 | 8.23E+09 |
| EPI_ISL_2226223 | Nasopharynx | 12.53 | 3.43E+09 | 11.35 | 7.76E+09 |
| EPI_ISL_2226224 | Sputum | 16.65 | 1.98E+08 | 16.34 | 2.46E+08 |
| EPI_ISL_2226225 | Nasopharynx | 15.58 | 9.35E+07 | 15.79 | 8.84E+07 |
| EPI_ISL_2226226 | Nasopharynx | 11.14 | 8.97E+09 | 10.15 | 8.48E+09 |
| EPI_ISL_2226227 | Nasopharynx | 13.21 | 4.26E+08 | 12.7 | 6.29E+08 |
| EPI_ISL_2226228 | Nasopharynx | 12.76 | 5.68E+08 | 11.68 | 1.20E+09 |

**Supplementary Table 2. NGS results for all SARS-COV-2 genomes isolated from COVID-19 patients**

| **Sample ID** | **Gene** | **Position** | **Reference amino acid** | | **Mutated amino acid** |
| --- | --- | --- | --- | --- | --- |
| EPI_ISL_985394 | ORF1a (NSP3) | 951 | Methionine | | Isoleucine |
|  | ORF1a (NSP6) | 37 | Leucine | | Phenylalanine |
|  | ORF3 (NS3) | 251 | Glycine | | Valine |
| EPI_ISL_1007662 | ORF1a (NSP3) | 951 | Methionine | | Isoleucine |
|  | ORF1a (NSP6) | 37 | Leucine | | Phenylalanine |
|  | Spike (S) | 655 | Histidine | | Tyrosine |
|  | ORF3 (NS3) | 251 | Glycine | | Valine |
| EPI_ISL_1007663 | ORF1a (NSP3) | 951 | Methionine | | Isoleucine |
|  | ORF1a (NSP6) | 37 | Leucine | | Phenylalanine |
|  | ORF3 (NS3) | 251 | Glycine | | Valine |
| EPI_ISL_1007664 | ORF1a (NSP3) | 951 | Methionine | | Isoleucine |
|  | ORF1a (NSP6) | 37 | Leucine | | Phenylalanine |
|  | ORF3 (NS3) | 251 | Glycine | | Valine |
| EPI_ISL_1007665 | ORF1a (NSP3) | 951 | Methionine | | Isoleucine |
|  | ORF1a (NSP6) | 37 | Leucine | | Phenylalanine |
|  | ORF3 (NS3) | 251 | Glycine | | Valine |
|  | Nucleocapsid(N) | 120 | Glycine | | Glutamic acid |
|  |  | 237 | Lysine | | Threonine |
| EPI_ISL_1007666 | ORF1a (NSP3) | 951 | Methionine | | Isoleucine |
|  | ORF1a (NSP6) | 37 | Leucine | | Phenylalanine |
|  | ORF3 (NS3) | 251 | Glycine | | Valine |
| EPI_ISL_1007667 | ORF1a (NSP3) | 951 | Methionine | | Isoleucine |
|  | ORF1a (NSP6) | 37 | Leucine | | Phenylalanine |
|  | ORF3 (NS3) | 251 | Glycine | | Valine |
| EPI_ISL_1007668 | ORF1a (NSP3) | 951 | Methionine | | Isoleucine |
|  | ORF1a (NSP6) | 37 | Leucine | | Phenylalanine |
|  | ORF3 (NS3) | 251 | Glycine | | Valine |
| EPI_ISL_1007669 | ORF1a (NSP3) | 951 | Methionine | | Isoleucine |
|  | ORF1a (NSP6) | 37 | Leucine | | Phenylalanine |
|  | ORF3 (NS3) | 251 | Glycine | | Valine |
| EPI_ISL_1007670 | ORF1a (NSP3) | 951 | Methionine | | Isoleucine |
|  | ORF1a (NSP6) | 37 | Leucine | | Phenylalanine |
|  | ORF3 (NS3) | 251 | Glycine | | Valine |
| EPI_ISL_1007671 | ORF1a (NSP3) | 951 | Methionine | | Isoleucine |
|  | ORF1a (NSP6) | 37 | Leucine | | Phenylalanine |
|  | ORF3 (NS3) | 251 | Glycine | | Valine |
|  | ORF8 (NS8) | 30 | Proline | | Serine |
| EPI_ISL_1007672 | ORF1a (NSP2) | 85 | | Threonine | Isoleucine |
|  | ORF1a (NSP7) | 25 | | Serine | Leucine |
|  | ORF1b (NSP12) | 323 | | Proline | Leucine |
|  | ORF1b (NSP16) | 6 | | Glutamine | Leucine |
|  | Spike (S) | 614 | | Aspartic acid | Glycine |
|  | ORF3 (NS3) | 57 | | Glutamine | Histidine |
| EPI_ISL_1007673 | ORF1a (NSP2) | 85 | | Threonine | Isoleucine |
|  | ORF1a (NSP7) | 25 | | Serine | Leucine |
|  | ORF1b (NSP12) | 323 | | Proline | Leucine |
|  | ORF1b (NSP16) | 6 | | Glutamine | Leucine |
|  | Spike (S) | 614 | | Aspartic acid | Glycine |
|  | ORF3 (NS3) | 57 | | Glutamine | Histidine |
|  | ORF7a | 84 | | Proline | Serine |
| EPI_ISL_1007674 | ORF1a (NSP2) | 85 | | Threonine | Isoleucine |
|  | ORF1a (NSP7) | 25 | | Serine | Leucine |
|  | ORF1a (NSP8) | 23 | | Glutamic acid | Valine |
|  | ORF1b (NSP12) | 323 | | Proline | Leucine |
|  | ORF1b (NSP14) | 119 | | Alanine | Valine |
|  | ORF1b (NSP16) | 6 | | Glutamine | Leucine |
|  | Spike (S) | 589 | | Proline | Serine |
|  | Spike (S) | 614 | | Aspartic acid | Glycine |
|  | ORF3 (NS3) | 57 | | Glutamine | Histidine |
|  | Envelope (E) | 5 | | Valine | Phenylalanine |
|  | ORF7a | 84 | | Proline | Serine |
| EPI_ISL_1007675 | ORF1a (NSP2) | 85 | | Threonine | Isoleucine |
|  | ORF1a (NSP3) | 103 | | Tyrosine | Cysteine |
|  |  | 113 | | Glutamic acid | Glycine |
|  | ORF1a (NSP4) | 438 | | Leucine | Arginine |
|  | ORF1a (NSP7) | 25 | | Serine | Leucine |
|  | ORF1b (NSP12) | 323 | | Proline | Leucine |
|  |  | 638 | | Leucine | Phenylalanine |
|  | ORF1b (NSP14) | 132 | | Aspartic acid | Tyrosine |
|  | ORF1b (NSP16) | 6 | | Glutamine | Leucine |
|  | Spike (S) | 180 | | Glutamic acid | Lysine |
|  |  | 614 | | Aspartic acid | Glycine |
|  | ORF3 (NS3) | 57 | | Glutamine | Histidine |
|  | Nucleocapsid(N) | 205 | | Threonine | Isoleucine |
| EPI_ISL_1007676 | ORF1a (NSP2) | 85 | | Threonine | Isoleucine |
|  | ORF1a (NSP3) | 113 | | Glutamic acid | Glycine |
|  | ORF1a (NSP7) | 25 | | Serine | Leucine |
|  | ORF1b (NSP12) | 323 | | Proline | Leucine |
|  |  | 638 | | Leucine | Phenylalanine |
|  | ORF1b (NSP16) | 6 | | Glutamine | Leucine |
|  | Spike (S) | 614 | | Aspartic acid | Glycine |
|  | ORF3 (NS3) | 57 | | Glutamine | Histidine |
| EPI_ISL_1007677 | ORF1a (NSP2) | 85 | | Threonine | Isoleucine |
|  | ORF1a (NSP3) | 1456 | | Threonine | Isoleucine |
|  | ORF1a (NSP7) | 25 | | Serine | Leucine |
|  | ORF1b (NSP12) | 323 | | Proline | Leucine |
|  | ORF1b (NSP16) | 6 | | Glutamine | Leucine |
|  | Spike (S) | 614 | | Aspartic acid | Glycine |
|  | ORF3 (NS3) | 2 | | Aspartic acid | Glycine |
|  |  | 57 | | Glutamine | Histidine |
|  | ORF8 (NS8) | 36 | | Proline | Serine |
| EPI_ISL_1007678 | ORF1a (NSP2) | 85 | | Threonine | Isoleucine |
|  | ORF1a (NSP3) | 1456 | | Threonine | Isoleucine |
|  | ORF1a (NSP7) | 25 | | Serine | Leucine |
|  | ORF1b (NSP12) | 323 | | Proline | Leucine |
|  | ORF1b (NSP16) | 6 | | Glutamine | Leucine |
|  | Spike (S) | 614 | | Aspartic acid | Glycine |
|  | ORF3 (NS3) | 57 | | Glutamine | Histidine |
|  | ORF8 (NS8) | 36 | | Proline | Serine |
| EPI_ISL_1007679 | ORF1a (NSP2) | 85 | | Threonine | Isoleucine |
|  | ORF1a (NSP3) | 295 | | Histidine | Tyrosine |
|  |  | 1365 | | Threonine | Isoleucine |
|  |  | 1590 | | Threonine | Asparagine |
|  | ORF1a (NSP4) | 204 | | Threonine | Isoleucine |
|  | ORF1a (NSP7) | 25 | | Serine | Leucine |
|  | ORF1a (NSP9) | 83 | | Proline | Serine |
|  | ORF1b (NSP12) | 153 | | Aspartic acid | Tyrosine |
|  |  | 323 | | Proline | Leucine |
|  | ORF1b (NSP13) | 446 | | Alanine | Serine |
|  | ORF1b (NSP16) | 6 | | Glutamine | Leucine |
|  | Spike (S) | 614 | | Aspartic acid | Glycine |
|  | ORF3 (NS3) | 57 | | Glutamine | Histidine |
|  |  | 213 | | Glutamine | Lysine |
|  | ORF7a | 47 | | Histidine | Asparagine |
| EPI_ISL_2226216 | ORF1a (NSP2) | 85 | | Threonine | Isoleucine |
|  | ORF1a (NSP7) | 25 | | Serine | Leucine |
|  | ORF1a (NSP9) | 24 | | Threonine | Isoleucine |
|  | ORF1b (NSP12) | 323 | | Proline | Leucine |
|  | ORF1b (NSP14) | 119 | | Alanine | Valine |
|  | ORF1b (NSP16) | 6 | | Glutamine | Leucine |
|  | Spike (S) | 589 | | Proline | Serine |
|  |  | 614 | | Aspartic acid | Glycine |
|  | ORF3 (NS3) | 57 | | Glutamine | Histidine |
|  | ORF7a | 84 | | Proline | Serine |
| EPI_ISL_2226217 | ORF1a (NSP2) | 85 | | Threonine | Isoleucine |
|  |  | 365 | | Arginine | Leucine |
|  | ORF1a (NSP3) | 114 | | Glutamic acid | Glycine |
|  | ORF1a (NSP4) | 204 | | Threonine | Isoleucine |
|  | ORF1a (NSP7) | 25 | | Serine | Leucine |
|  | ORF1b (NSP12) | 323 | | Proline | Leucine |
|  | ORF1b (NSP16) | 6 | | Glutamine | Leucine |
|  | Spike (S) | 614 | | Aspartic acid | Glycine |
|  | ORF3 (NS3) | 57 | | Glutamine | Histidine |
|  |  | 174 | | Glycine | Cysteine |
| EPI_ISL_2226218 | ORF1a (NSP2) | 85 | | Threonine | Isoleucine |
|  |  | 365 | | Arginine | Leucine |
|  | ORF1a (NSP3) | 114 | | Glutamic acid | Glycine |
|  | ORF1a (NSP4) | 204 | | Threonine | Isoleucine |
|  | ORF1a (NSP7) | 25 | | Serine | Leucine |
|  | ORF1b (NSP12) | 323 | | Proline | Leucine |
|  | ORF1b (NSP16) | 6 | | Glutamine | Leucine |
|  | Spike (S) | 614 | | Aspartic acid | Glycine |
|  | ORF3 (NSP3) | 57 | | Glutamine | Histidine |
| EPI_ISL_2226219 | ORF1a (NSP2) | 85 | | Threonine | Isoleucine |
|  | ORF1a (NSP3) | 114 | | Glutamic acid | Glycine |
|  | ORF1a (NSP7) | 25 | | Serine | Leucine |
|  | ORF1b (NSP12) | 323 | | Proline | Leucine |
|  | ORF1b (NSP16) | 6 | | Glutamine | Leucine |
|  | Spike (S) | 614 | | Aspartic acid | Glycine |
|  | ORF3 (NS3) | 57 | | Glutamine | Histidine |
| EPI_ISL_2226220 | ORF1a (NSP2) | 85 | | Threonine | Isoleucine |
|  | ORF1a (NSP3) | 1456 | | Threonine | Isoleucine |
|  | ORF1a (NSP7) | 25 | | Serine | Leucine |
|  | ORF1b (NSP12) | 323 | | Proline | Leucine |
|  | ORF1b (NSP16) | 6 | | Glutamine | Leucine |
|  | Spike (S) | 614 | | Aspartic acid | Glycine |
|  | ORF3 (NS3) | 57 | | Glutamine | Histidine |
| EPI_ISL_2226221 | ORF1a (NSP2) | 85 | | Threonine | Isoleucine |
|  | ORF1a (NSP3) | 1456 | | Threonine | Isoleucine |
|  | ORF1a (NSP7) | 25 | | Serine | Leucine |
|  | ORF1b (NSP12) | 323 | | Proline | Leucine |
|  | ORF1b (NSP16) | 6 | | Glutamine | Leucine |
|  | Spike (S) | 245 | | Histidine | Arginine |
|  |  | 614 | | Aspartic acid | Glycine |
|  |  | 686 | | Serine | Isoleucine |
|  | ORF3 (NS3) | 57 | | Glutamine | Histidine |
| EPI_ISL_2226222 | ORF1a (NSP7) | 25 | | Serine | Leucine |
|  | ORF1b (NSP12) | 323 | | Proline | Leucine |
|  |  | 335 | | Valine | Isoleucine |
|  | ORF1b (NSP16) | 6 | | Glutamine | Leucine |
|  |  | 158 | | Glutamine | Lysine |
|  | Spike (S) | 614 | | Aspartic acid | Glycine |
|  |  | 623 | | Alanine | Serine |
|  | ORF3 (NS3) | 57 | | Glutamine | Histidine |
| EPI_ISL_2226223 | ORF1a (NSP2) | 85 | | Threonine | Isoleucine |
|  | ORF1a (NSP3) | 1456 | | Threonine | Isoleucine |
|  | ORF1a (NSP7) | 25 | | Serine | Leucine |
|  | ORF1b (NSP12) | 323 | | Proline | Leucine |
|  | ORF1b (NSP16) | 6 | | Glutamine | Leucine |
|  | Spike (S) | 614 | | Aspartic acid | Glycine |
|  | ORF3 (NS3) | 57 | | Glutamine | Histidine |
| EPI_ISL_2226224 | ORF1a (NSP2) | 85 | | Threonine | Isoleucine |
|  |  | 530 | | Valine | Leucine |
|  |  | 531 | | Threonine | Proline |
|  | ORF1a (NSP3) | 1456 | | Threonine | Isoleucine |
|  | ORF1a (NSP7) | 25 | | Serine | Leucine |
|  | ORF1b (NSP12) | 323 | | Proline | Leucine |
|  | ORF1b (NSP16) | 6 | | Glutamine | Leucine |
|  | Spike (S) | 614 | | Aspartic acid | Glycine |
|  | ORF3 (NS3) | 57 | | Glutamine | Histidine |
| EPI_ISL_2226225 | ORF1a (NSP2) | 85 | | Threonine | Isoleucine |
|  | ORF1a (NSP3) | 1456 | | Threonine | Isoleucine |
|  | ORF1a (NSP7) | 25 | | Serine | Leucine |
|  | ORF1b (NSP12) | 323 | | Proline | Leucine |
|  | ORF1b (NSP16) | 6 | | Glutamine | Leucine |
|  | Spike (S) | 614 | | Aspartic acid | Glycine |
|  | ORF3 (NS3) | 57 | | Glutamine | Histidine |
| EPI_ISL_2226226 | ORF1a (NSP2) | 85 | | Threonine | Isoleucine |
|  | ORF1a (NSP3) | 1456 | | Threonine | Isoleucine |
|  | ORF1a (NSP7) | 25 | | Serine | Leucine |
|  | ORF1b (NSP12) | 323 | | Proline | Leucine |
|  | ORF1b (NSP16) | 6 | | Glutamine | Leucine |
|  | Spike (S) | 68 | | Isoleucine | Lysine |
|  |  | 614 | | Aspartic acid | Glycine |
|  | ORF3 (NS3) | 57 | | Glutamine | Histidine |
| EPI_ISL_2226227 | ORF1a (NSP2) | 85 | | Threonine | Isoleucine |
|  | ORF1a (NSP3) | 295 | | Histidine | Tyrosine |
|  |  | 1590 | | Threonine | Asparagine |
|  | ORF1a (NSP7) | 25 | | Serine | Leucine |
|  | ORF1a (NSP9) | 83 | | Proline | Serine |
|  | ORF1b (NSP12) | 323 | | Proline | Leucine |
|  | ORF1b (NSP13) | 446 | | Alanine | Serine |
|  | ORF1b (NSP16) | 6 | | Glutamine | Leucine |
|  | Spike (S) | 52 | | Glutamine | Histidine |
|  |  | 556 | | Asparagine | Lysine |
|  |  | 614 | | Aspartic acid | Glycine |
|  | ORF3 (NS3) | 57 | | Glutamine | Histidine |
| EPI_ISL_2226228 | ORF1a (NSP2) | 85 | | Threonine | Isoleucine |
|  | ORF1a (NSP3) | 295 | | Histidine | Tyrosine |
|  |  | 1590 | | Threonine | Asparagine |
|  | ORF1a (NSP7) | 25 | | Serine | Leucine |
|  | ORF1a (NSP9) | 83 | | Proline | Serine |
|  | ORF1b (NSP12) | 323 | | Proline | Leucine |
|  | ORF1b (NSP13) | 446 | | Alanine | Serine |
|  | ORF1b (NSP16) | 6 | | Glutamine | Leucine |
|  | Spike (S) | 52 | | Glutamine | Histidine |
|  |  | 556 | | Asparagine | Lysine |
|  |  | 614 | | Aspartic acid | Glycine |
|  | ORF3 (NS3) | 57 | | Glutamine | Histidine |

**Supplementary Table 3. Mutation linkage analysis showing the mutation positions and the number of isolates/patients in which those mutations were observed at different time point**

| **Mutation position** | **Sample collection time** | **Number of isolates** | **Number of patients** |
| --- | --- | --- | --- |
| ORF1a (nsp3) - M951I | First wave (February to May 2020) | 11 | 4 |
| ORF1a (nsp6) - L37F |  | 11 | 4 |
| ORF3 (ns3) - G251V |  | 11 | 4 |
| Spike (S) - H655Y |  | 1 | 1 |
| Nucleocapsid(N) - G120E |  | 1 | 1 |
| Nucleocapsid(N) - K237T |  | 1 | 1 |
| ORF8 (ns8) - P30S |  | 1 | 1 |
| ORF1a (nsp2) - T85I | Second wave (June 2020 to April 2021) | 20 | 14 |
| ORF1a (nsp7) - S25L |  | 21 | 14 |
| ORF1b (nsp12) - P323L |  | 21 | 14 |
| ORF1b (nsp16) - Q6L |  | 21 | 14 |
| Spike (S) - D614G |  | 21 | 14 |
| ORF3 (ns3) - Q57H |  | 21 | 14 |
| ORF7a - P84S |  | 3 | 3 |
| ORF1a (nsp8) - E23V |  | 1 | 1 |
| ORF1b (nsp14) - A119V |  | 2 | 2 |
| Spike (S) - P589S |  | 2 | 2 |
| Envelope E - V5F |  | 1 | 1 |
| ORF1a (nsp3) - Y103C |  | 1 | 1 |
| ORF1a (nsp3) - E113G |  | 2 | 2 |
| ORF1a (nsp4) - L438R |  | 1 | 1 |
| ORF1b (nsp12) - L638F |  | 2 | 2 |
| ORF1b (nsp14) - D132Y |  | 1 | 1 |
| Spike (S) - E180K |  | 1 | 1 |
| Nucleocapsid(N) - T205I |  | 1 | 1 |
| ORF1a (nsp3) - T1456I |  | 8 | 3 |
| Spike (S) - H245R |  | 1 | 1 |
| Spike (S) - S686I |  | 1 | 1 |
| Spike (S) - I68K |  | 1 | 1 |
| ORF1a (nsp3) - H295Y |  | 3 | 2 |
| ORF1a (nsp3) - T1590N |  | 3 | 2 |
| ORF1a (nsp9) - P83S |  | 3 | 2 |
| ORF1b (nsp13) - A446S |  | 3 | 2 |
| Spike (S) - Q52H |  | 2 | 1 |
| Spike (S) - N556K |  | 2 | 1 |
| ORF8 (ns8) - P36S |  | 2 | 2 |
| ORF1a (nsp3) - T1365I |  | 1 | 1 |
| ORF1a (nsp4) - T204I |  | 3 | 3 |
| ORF1b (nsp12) - D153Y |  | 1 | 1 |
| ORF3 (ns3) - Q213K |  | 1 | 1 |
| ORF7a - H47N |  | 1 | 1 |
| ORF1a (nsp9) - T24I |  | 1 | 1 |
| ORF1a (nsp2) - R365L |  | 2 | 2 |
| ORF3 (ns3) - G174C |  | 1 | 1 |
| ORF1b (nsp12) - V335I |  | 1 | 1 |
| ORF1b (nsp16) - Q158K |  | 1 | 1 |
| ORF1a (nsp2) - V530L |  | 1 | 1 |
| ORF1a (nsp2) - T531P |  | 1 | 1 |
| Spike (S) - A623S |  | 1 | 1 |
| ORF3 (ns3) - D2G |  | 1 | 1 |
| ORF1a (nsp3) - E114G |  | 3 | 3 |
